# A high‐throughput biomimetic bone‐on‐a‐chip platform with artificial intelligence‐assisted image analysis for osteoporosis drug testing

**DOI:** 10.1002/btm2.10313

**Published:** 2022-04-05

**Authors:** Kyurim Paek, Seulha Kim, Sungho Tak, Min Kyeong Kim, Jubin Park, Seok Chung, Tai Hyun Park, Jeong Ah Kim

**Affiliations:** ^1^ Center for Scientific Instrumentation Korea Basic Science Institute Daejeon South Korea; ^2^ Program in Micro/Nano System Korea University Seoul South Korea; ^3^ School of Chemical and Biological Engineering, Institute of Chemical Processes Seoul National University Seoul South Korea; ^4^ Research Center for Bioconvergence Analysis Korea Basic Science Institute Cheongju Chungbuk South Korea; ^5^ School of Mechanical Engineering Korea University Seoul South Korea; ^6^ Department of Bio‐Analytical Science University of Science and Technology Daejeon South Korea

**Keywords:** bone formation, bone‐on‐a‐chip, deep learning, drug testing, high‐throughput analysis, osteoporosis

## Abstract

Although numerous organ‐on‐a‐chips have been developed, bone‐on‐a‐chip platforms have rarely been reported because of the high complexity of the bone microenvironment. With an increase in the elderly population, a high‐risk group for bone‐related diseases such as osteoporosis, it is essential to develop a precise bone‐mimicking model for efficient drug screening and accurate evaluation in preclinical studies. Here, we developed a high‐throughput biomimetic bone‐on‐a‐chip platform combined with an artificial intelligence (AI)‐based image analysis system. To recapitulate the key aspects of natural bone microenvironment, mouse osteocytes (IDG‐SW3) and osteoblasts (MC3T3‐E1) were cocultured within the osteoblast‐derived decellularized extracellular matrix (OB‐dECM) built in a well plate‐based three‐dimensional gel unit. This platform spatiotemporally and configurationally mimics the characteristics of the structural bone unit, known as the osteon. Combinations of native and bioactive ingredients obtained from the OB‐dECM and coculture of two types of bone cells synergistically enhanced osteogenic functions such as osteocyte differentiation and osteoblast maturation. This platform provides a uniform and transparent imaging window that facilitates the observation of cell–cell interactions and features high‐throughput bone units in a well plate that is compatible with a high‐content screening system, enabling fast and easy drug tests. The drug efficacy of anti‐SOST antibody, which is a newly developed osteoporosis drug for bone formation, was tested via β‐catenin translocation analysis, and the performance of the platform was evaluated using AI‐based deep learning analysis. This platform could be a cutting‐edge translational tool for bone‐related diseases and an efficient alternative to bone models for the development of promising drugs.

## 1 INTRODUCTION

Bone is a rigid but dynamic tissue that performs essential functions in the body, such as structural support, mobility, endocrine regulation, and mineral storage.^1^, ^2^ It is continuously remodeled through highly coordinated actions of several types of bone cells, such as osteocytes, osteoblasts, and osteoclasts. Any imbalance in this orchestrated process among these cells can disrupt normal bone homeostasis, leading to severe diseases.^1^, ^3^, ^4^, ^5^ Osteoporosis is one of the most common diseases frequently occurring in older women over 50 years of age, which results from an imbalance between bone formation and bone resorption.^1^, ^4^, ^5^, ^6^ Despite the importance of bone, the ability to investigate processes related to either physiologic or diseased bone tissue has been hindered by traditional models that fail to emulate the complexity of bone.^2^, ^7^, ^8^

In the abovementioned context, it is crucial to develop an efficient biomimetic system that simulates the three‐dimensional (3D) nature of the bone microenvironment along with the dynamic cell–cell and cell–matrix interactions.^7^, ^9^, ^10^, ^11^ The Transwell system, a representative in vitro cell culture platform that can accommodate different types of cells to induce cell–cell interactions, still has limitations in recapitulating the physiological characteristics of bone because of the unrealistically long distance between cells owing to the vertically arranged chamber structure, which makes it difficult to monitor and analyze cells in different focal planes.^12^ Recently, functional 3D bone tissues based on artificial scaffolds, such as synthetic polymers,^8^, ^13^ metals,^8^, ^14^, ^15^, ^16^, ^17^ and ceramics,^8^, ^18^ have been suggested. However, the existing models include limitations of large dead volume, difficulty in observing samples optically, and limited availability of high‐throughput analysis.^18^, ^19^

With technological advances in microfluidics and tissue engineering, the development of an organ‐on‐a‐chip that enables the reproduction of key elements in tissue‐specific microenvironments has been suggested. Compared with other conventional 2D in vitro or animal models, organ‐on‐a‐chip platforms are physiologically relevant because they provide a controlled spatiotemporal environment as well as hydrodynamic stimuli, such as fluid and mechanical cues.^20^, ^21^ In addition, these platforms provide increased predictive power for clinical assays using miniaturized and high‐throughput techniques combined with large‐scale integration and automatic operation systems.^20^, ^22^

During the last decade, many organs‐on‐chips mimicking various organs such as the liver, lung, brain, kidney, heart, and intestine have been investigated in many studies; however, bone models based on chip platforms have rarely been reported.^7^, ^20^ This can be attributed to the failure in developing a model that explicitly recapitulates the complex biology of bone tissues. Bone tissue is characterized by a complex structural arrangement of different types of cells, including osteocytes embedded in mineralized organic matrix and osteoblasts located on the bone surface.^1^, ^2^ Another limitation is that it is difficult to obtain and culture human primary bone cells; hence, many studies have used animal‐derived cells.^23^, ^24^ Moreover, previously established bone models still rely on combining scaffolds with tissue‐derived matrix and complex materials.^8^, ^18^, ^25^ These approaches are unfavorable for high‐throughput screening for preclinical use.^19^, ^26^, ^27^

In this study, we developed a high‐throughput biomimetic bone‐on‐a‐chip platform that recapitulates the physiologically functional bone unit, called osteon, using a well plate‐based system. First, we mimicked the native bone microenvironment using an osteoblast‐derived decellularized extracellular matrix (ECM) (OB‐dECM) with high bioactivity as an embedding matrix for osteocytes to enhance cell–ECM interactions. Second, we mimicked the configurational features of osteons by utilizing the unique design of our platform consisting of circular chambers with two compartments. This orientation is favorable for inducing cell–cell interactions. The functional feasibility of our bone model was demonstrated in terms of differentiation, proliferation, and interactions between osteoblasts and osteocytes using various biochemical assays. Finally, we demonstrated whether this highly orchestrated bone‐on‐a‐chip platform could be used for testing osteoporosis drugs. As a proof‐of‐concept, the anti‐SOST antibody drug, which can regulate the Wnt signaling pathway and promote osteoblast growth,^5^, ^28^, ^29^ was tested using this chip system, and its efficacy was evaluated using an intelligent and powerful artificial intelligence (AI)‐based image analysis system. This integrated system will accelerate significant advances in bone model development, help understand bone physiology and bone‐related diseases, and expand its applicability in the preclinical stage for drug development.

## 2 MATERIALS AND METHODS

### 2.1 Fabrication of devices for the bone‐on‐a‐chip

Bone‐on‐a‐chip devices were fabricated using well plate‐based hydrogel‐incorporating gel units, as described previously,^30^ and are advantageous for a variety of cell biology applications and high‐throughput analysis. The chip design and microfabrication process are depicted in Figure S1A,B. In summary, the Sylgard 184 elastomer, polydimethylsiloxane (PDMS) (Dow Corning) was poured onto the mold, fabricated using a photolithography method, having a thickness of approximately 2 mm. After polymerization, the PDMS replicas were peeled off and the inlet hole (1‐mm diameter) for injecting the gel was punched out. The dumbbell‐shaped outline of the chip was cut using a customized punch. To accurately position the PDMS replica in the center of the well plate, a customized jig made of polyether ether ketone using machining was employed (Figure S1C). The PDMS replicas were mounted on the jig and plasma‐treated. Finally, a glass bottom 24‐well plate (Mattek) was inverted and placed on the jig. Immediately after bonding, the inner surfaces of the chip were coated with 1 mg/ml of dopamine hydrochloride solution prepared in phosphate‐buffered saline (PBS) (Sigma‐Aldrich) for 1.5 h to enhance hydrogel adhesion on the chip surface and washed thoroughly five times with DW. The devices were dried in an oven at 80°C.

### 2.2 Cell culture

Mouse osteocyte‐like cells, IDG‐SW3 (Kerafast), were maintained in collagen‐coated flasks containing alpha minimum essential medium (Alpha‐MEM; Gibco) supplemented with 10% (vol/vol) heat‐inactivated FBS (Gibco), 50 U/ml of recombinant mouse protein interferon‐γ (IFN‐γ) (Gibco), and 1% (wt/vol) penicillin/streptomycin (Thermo Fisher Scientific) at 33°C in a 5% (vol/vol) CO_2_ incubator. Mouse preosteoblasts, MC3T3‐E1 cells (ATCC), were maintained in alpha‐MEM supplemented with 10% fetal bovine serum and 1% (wt/vol) penicillin/streptomycin at 37°C in a 5% (vol/vol) CO_2_ incubator. Cells were detached using 0.25% (wt/vol) trypsin–EDTA (Gibco) when they reached 80% confluency. The complete medium was exchanged every 2–3 days. For osteogenic differentiation (late osteoblast to osteocyte) of IDG‐SW3 cells, cells were cultured in a differentiation medium (alpha‐MEM) supplemented with 10% (vol/vol) FBS, 50 μg/ml of ascorbic acid, 4 mM β‐glycerophosphate, and 1% (wt/vol) penicillin/streptomycin lacking IFN‐γ at 37°C. MC3T3‐E1 cells were differentiated (preosteoblasts to osteoblasts) following the same process.

### 2.3 Preparation of OB‐dECM

OB‐dECM was extracted from MC3T3‐E1 cells that were cultured for 14 days and then redissolved (8 mg/ml) according to the established process described in a previous paper.^31^ The lyophilized OB‐dECM was chopped and mixed with 1 mg/ml pepsin (Sigma‐Aldrich) in 0.1 M acetic acid solution to ensure enzymatic hydrolysis by stirring at 500 rpm for 12 h at 4°C using a magnetic bar. The pepsin present in the OB‐dECM solution was inactivated by adding 50 μg/ml of pepstatin (Sigma‐Aldrich), and the solution was stored at 4°C until further use (Figure S2A). Rat tail collagen type I (3–4 mg/ml; Corning) and OB‐dECM were mixed to form a Col/OB‐dECM composite hydrogel. The optimized composition of the hydrogel was 2 mg/ml collagen and 1 mg/ml OB‐dECM in the hydrogel solution. For gelation, 0.5 M sodium hydroxide was used for neutralization, and the osmotic pressure of the hydrogel was adjusted using 10X PBS.

### 2.4 Preparation of bone‐on‐a‐chip system

IDG‐SW3 and MC3T‐E1 cells were cultured in the device, as depicted in Figure S2B. IDG‐SW3 cells (1 × 10^6^ cells/ml) mixed with Col/OB‐dECM were embedded in the 3D hydrogel and injected into the chip. After gelation, MC3T3‐E1 cells (5 × 10^2^ cells) were seeded into the margined region surrounding the chip in a well. For osteogenic differentiation, cells were cultured in an osteogenic medium, and the medium was replaced every 2 days.

### 2.5 Mechanical characterization of hydrogels

The hydrogel was prepared in a PDMS mold (10 mm in diameter, 5 mm in height) and maintained in 1X Hank's balanced salt solution at 37°C overnight before measurement. The hydrogel was placed on a stainless spatula, and the excess buffer around the spatula was removed. For determining the Young's modulus, the change in the compressive pressure (force/area) along with the ratio of change in hydrogel height (Δheight/height) was measured using a tensile tester (EZ‐SX; Shimadzu). Considering that the contact area between the hydrogel and tester increases while being pressed, only the slope of the initial linear section was taken and calculated according to the following equation: Young's modulus = (force/area)/(∆height/height).

The viscoelastic properties of the hydrogels were measured using a stress‐controlled rheometer (AR‐G2; TA Instruments) with a 10‐mm steel plate. The rheometer gap was 850–1000 μm. A strain sweep was performed to confirm the linear elastic regime. The storage modulus (*G*′) of the hydrogels was measured using an oscillatory frequency sweep conducted at 0.5% strain in the frequency range of 0.62–10 Hz.

### 2.6 Cell viability measurement

The viability of 3D cultured IDG‐SW3 cells in the hydrogel was assessed using a CellTiter‐Glo 3D Cell Viability Assay kit (Promega). Briefly, equal volumes of the reagent and cell culture medium were added to each well. Cells were lysed by vigorous mixing for 60 min. After complete lysis of the cells, luminescence was recorded using a microplate reader (Spark; Tecan).

### 2.7 Alkaline phosphatase activity assay

Alkaline phosphatase (ALP) activity in IDG‐SW3 cells 3D cultured in a bone‐on‐a‐chip using an ALP Activity Assay Kit (Cell Biolabs) on Days 7 and 14 after differentiation. The quantitative absorbance was measured at 405 nm using a spectrophotometer (SoftMax Pro 5; Molecular Devices).

### 2.8 Quantitative real‐time polymerase chain reaction

Quantitative real‐time polymerase chain reaction (qRT‐PCR) was performed to analyze the mRNA levels of *ALP*, phosphate‐regulating endopeptidase homolog X‐linked (*PHEX*), podoplanin (*PDPN*), dentin matrix acidic phosphoprotein 1 (*DMP*1), sclerostin (*SOST*), and fibroblast growth factor 23 (*FGF23*) in IDG‐SW3 cells, and the mRNA levels of *cyclin D1*, *c‐Myc*, catenin beta 1 (*CTNNB1*), runt‐related transcription factor 2 (*RUNX2*), osterix (*Osx*), and osteoprotegerin (*OPG*) in MC3T3‐E1 cells. Total mRNA was extracted from cells using an RNeasy Mini Kit (Qiagen). Eighty nanograms of total RNA from IDG‐SW3 cells and 200 ng of total RNA from MC3T3‐E1 cells were reverse transcribed using a ReverTra Ace qPCR RT Master Mix with gDNA Remover Kit (Toyobo). *GAPDH* was used as an internal control. PCR was performed using a miScript SYBR Green PCR kit (Qiagen) with 40 cycles on a PCR machine (QuantStudio 3; Thermo Fisher Scientific). Primer sequences are listed in Table S1.

### 2.9 Immunocytochemistry

IDG‐SW3 cells in hydrogel and MC3T3‐E1 cells were fixed with 4% (vol/vol) paraformaldehyde for 20 min, permeabilized for 15 min using 0.01% (vol/vol) Triton X‐100 (Sigma‐Aldrich), and blocked with 5% bovine serum albumin in PBS containing 0.1% (wt/vol) Triton X‐100 for 30 min at room temperature. Live MC3T3‐E1 cells were stained with CellTrace Calcein red‐orange AM (Invitrogen). Cells were immunostained for F‐actin with Alexa Fluor 594‐conjugated phalloidin (1:400; Invitrogen) and for β‐catenin with Alexa Fluor 488‐conjugated β‐catenin antibody (1:50; Santa Cruz Biotechnology). Nuclei were stained with Hoechst 33342 (1:500; Thermo Fisher Scientific). Fluorescence images were obtained using a fluorescence microscope (Celena X High Content Imaging System; Logos Biosystems) and a confocal microscope (LSM‐710; Carl Zeiss).

### 2.10 Nuclear shape analysis

We analyzed the nuclear shape index (NSI) and nuclear alignment angles of IDG‐SW3 cells in the region of interest (ROI) (*n* = total number of cells in the ROI). A representative ROI was selected from the fluorescent cell images in the chip obtained using a confocal microscope (LSM‐710; Carl Zeiss). The areas and perimeters of the nucleus were measured using the ImageJ software, and the NSI was calculated from the relationship, NSI = 4π × area/perimeter. The NSI values ranged from 1 (circular shape) to 0 (elongated and linear). The nuclear alignment angles, defined as the direction of the long elliptical axis of the nucleus relative to the horizontal axis, were evaluated using the same software.

### 2.11 Cell counting kit proliferation assay

MC3T3‐E1 cell proliferation was measured using the Cell Counting Kit‐8 (CCK‐8) (DonginLS). After coculture, the chips containing IDG‐SW3 were removed from each well, mixed with 20 μl of CCK reagent and 200 μl of medium, and incubated for 30 min. The absorbance was recorded with a DTX 880 Multimode Detector (Beckman Coulter) at 450 nm. Cell proliferation was checked every 3 days from Day 1 to Day 10.

### 2.12 Enzyme‐linked immunosorbent assay

For mouse SOST ELISA, the conditioned medium of IDG‐SW3 cells cultured within the hydrogel in a chip was collected every 2–3 days for 2 weeks. The protein levels of SOST were measured using a mouse SOST ELISA Kit (ALPCO). In the case of connexin 43 (Cx43) ELISA, IDG‐SW3 cells were cultured for 7 and 14 days in the chip and lysed using a sonicator (GE‐50; Sonics & Materials), and the supernatant was used to measure the Cx43 protein level using a mouse Cx43 ELISA kit (MyBioSource).

### 2.13 Osteoporosis drug testing and image analysis

After culturing IDG‐SW3 cells in OB‐dECM hydrogel inside the chip for 10 days, MC3T3‐E1 cells were seeded and cocultured, and cells were treated with 20 ng/ml of anti‐SOST monoclonal antibody (Clone AbD09097_h/mIgG2a; Bio‐Rad), used as an osteoporosis drug,^32^ on the same day. The intensities and nucleus translocation rates of β‐catenin in MC3T3‐E1 cells were measured by building a pipeline using CellProfiler‐based modules (Celena X cell analyzer; Logos Biosystems) (*N* = the number of independent wells, three images per well were used to average). For pipeline sequences, the IdentifyPrimaryObjects module (identification of the location of the nucleus and β‐catenin), MaskImage module (segmentation of β‐catenin overlapped with the nucleus), and MeasureObjectIntensity module (determination of the intensity of β‐catenin in the whole and overlapping portions) were used. β‐Catenin nuclear translocation rates (Figure S3) were calculated using the following formula: β‐catenin nuclear translocation rates = *I*_n_/*I*_t_ × 100 (*I*_t_, β‐catenin intensity in the cell; *I*_n,_ β‐catenin intensity in the nucleus).

### 2.14 Deep learning algorithm

The image database containing β‐catenin, nucleus, and merged fluorescent images acquired from the bone‐on‐a‐chip platform was used for deep learning‐based image analysis. There were two labeled groups in the image data: the drug‐treated group (420 images) and the nondrug group (424 images). For data augmentation, one image was segmented into four images, and each image was randomly transformed using random zoom (zoom range of 0.5) and horizontal flip. For an algorithm classifying the images by group, a convolutional neural network (CNN) was used with fully connected layers. The entire network architecture consists of three convolution layers, three pooling layers, a dropout layer, a flattened layer, and two fully connected layers, as shown in Table S2. The 2D convolution layer (Conv2D) extracted features from the input images and generated a feature map. Conv2D was followed by rectified linear unit (ReLU) activation. For feature extraction, 2D max‐pooling layers (MaxPooling2D) extracted the maximum value from the feature map. In addition, dropout layers were applied randomly to a neural network during the training process to prevent overfitting. The flattened layer converts the features of the extracted data into 1D data. Finally, the classification algorithm was completed by connecting all the virtual neurons of the previous layers through two fully connected layers. Images corresponding to 10% of the total data were randomly selected and treated as the test set. The remaining images were used as a training set. The test set was used to evaluate the final performance of the classification algorithm. During the training process, a 10‐fold cross‐validation method was used to test network performance. The accuracy and area under the receiver operating characteristic curve (AUC ROC) were measured to evaluate the diagnostic ability of this algorithm using the dataset. ROC curves were created by plotting the sensitivity and the false positive rate (1‐specificity) based on the detection probability obtained by the classification algorithm.

### 2.15 Statistical analysis

All experiments were performed at least three times, and all numerical data were expressed as mean ± SD. Statistical significance was determined using a two‐tailed Student's *t*‐test. Statistical significance was denoted as *p < 0.05, **p < 0.01, ***p < 0.001, and ****p < 0.0001.

## 3 RESULTS AND DISCUSSION

### 3.1 A biomimetic bone‐on‐a‐chip platform based on a well plate for osteoporosis drug testing

**FIGURE 1:**
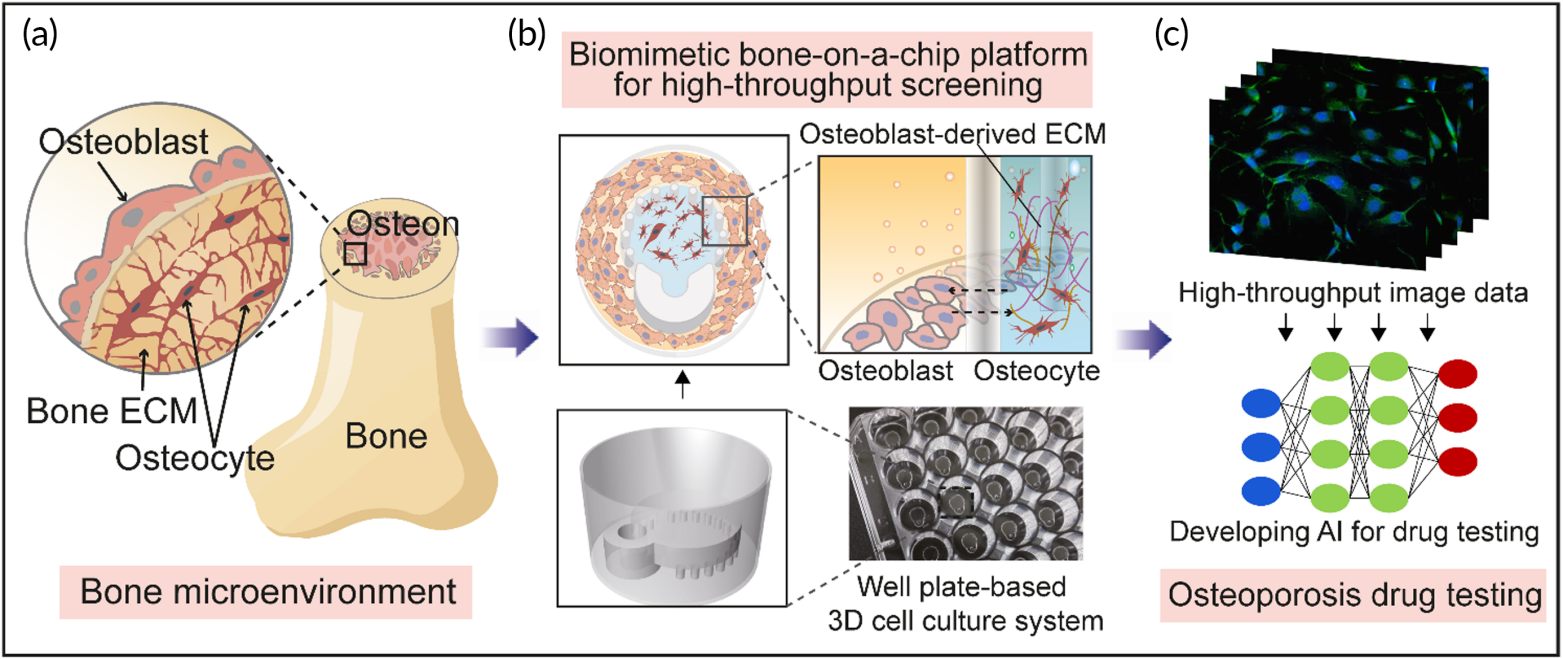
Schematic overview of a biomimetic bone‐on‐a‐chip platform combined with AI‐assisted image analysis for high‐throughput drug testing. (a) Illustration of 3D osteon niche of bone in vivo. (b) The configurational mimicking of bone in the biomimetic bone‐on‐a‐chip platform. Immature osteocytes were embedded in collagen and OB‐dECM composite (Col/OB‐dECM) within a chip, and preosteoblasts were cocultured in a region around the chip. Both cells were differentiated and matured to bone in bone‐on‐a‐chips built in a well plate. (c) Illustration of osteoporosis drug testing based on this platform and image data analysis using deep learning algorithms. 3D, three‐dimensional; AI, artificial intelligence; OB‐dECM, osteoblast‐derived decellularized extracellular matrix

Bone is a well‐organized tissue consisting of osteocytes, osteoblasts, and osteoclasts. Osteons, which are the functional units of bone, have a unique structural feature in which the osteocytes are embedded in the bone ECM while the osteoblasts surround and support them (Figure 1a). In this study, we used two strategies to develop a biomimetic bone‐on‐a‐chip platform to recapitulate the architectural and biochemical characteristics of the native bone niche. First, we employed several components of bone, such as osteocytes, osteoblasts, and OB‐dECM, to recreate the bone microenvironment. Similar to in vivo osteons, osteocytes were embedded in collagen gel composited with OB‐dECM (hereafter Col/OB‐dECM). Second, a 3D microstructured gel unit was used to create an orchestrated bone with these components. Our well plate‐based 3D cell culture device (Figure S1), which we previously devised and reported,^30^ can incorporate a cell‐laden hydrogel. This platform is a well plate‐based microfluidic channel‐free 3D cell culture device, which provides ease of handling of liquid and good compatibility with the microplate reader.^30^ The device also has an architecturally analogous feature for mimicking the osteon. The circular chamber of a gel unit can incorporate an osteocyte‐laden hydrogel in the center, and the surrounding region is suitable for accommodating neighboring cells, such as osteoblasts, enabling direct interaction among the cells (Figure 1b). Furthermore, this platform consists of uniform and multiple bone chips integrated into the well plate that is compatible with HTS and generates high‐throughput image data, which can be used for drug testing in combination with AI data analysis (Figure 1c). Using a soft‐lithography fabrication and bonding process with a custom‐made jig (Figure S1B,C), a consistent quality of chip platform was generated with an accurate position in a well, which made the procurement of high‐throughput image data easier and repeatable.

The rationale behind our study is that OB‐dECM could provide a functional biochemical factor to create a biomimetic bone because the native bone ECM is one of the key elements of the bone matrix. In addition, strong cell interactions can be induced through the horizontal arrangement of osteocytes and osteoblasts in the bone‐on‐a‐chip system. In the following sections, we demonstrate the biomimetic characteristics of the bone‐on‐a‐chip system and whether this system could be utilized for drug validation tests for osteoporosis, as a proof‐of‐concept.

### 3.2 Optimization and characterization of cell‐laden hydrogel in a bone‐on‐a‐chip

Bone tissue is composed of compact units called osteons, which are cylindrical structures composed of a central canal surrounded by a number of concentrically arranged mineralized bone matrices.^8^, ^31^ Osteocytes are housed and arranged within these osteons.

**FIGURE 2:**
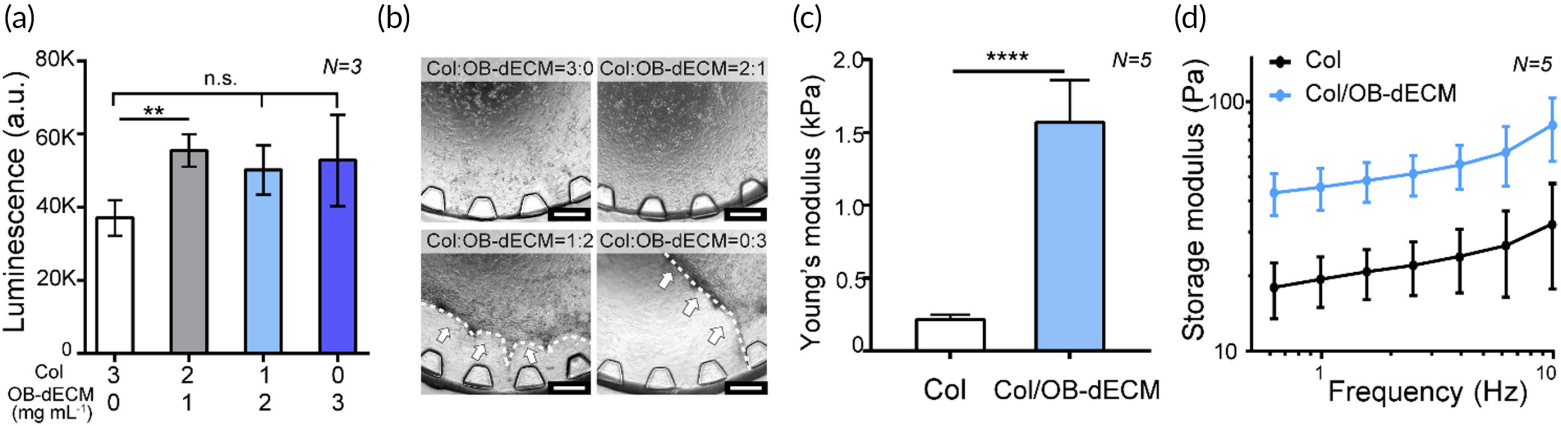
Optimization and characterization of cell‐laden OB‐dECM hydrogel in a bone‐on‐a‐chip. (a) Viability of IDG‐SW3 cells cultured in a hydrogel with different collagen/OB‐dECM ratios on Day 3 (*N* = 3). (b) Representative microscopic images of hydrogel shrinkage (white dotted line with arrows) according to collagen/OB‐dECM ratios on Day 3. Scale bar represents 500 μm. (c) Young's modulus and (d) storage modulus of the optimized Col/OB‐dECM hydrogel compared to the collagen hydrogel (*N* = 5). All values are expressed as mean ± SD (**p < 0.01, ****p < 0.0001). OB‐dECM, osteoblast‐derived decellularized extracellular matrix

Collagen is the most abundant ECM protein in bones,^2^, ^3^, ^8^ but it is still limited to mature bone owing to the lack of other ECM components in natural bone. In this study, we added OB‐dECM, which is abundantly secreted from osteoblasts, in the gel matrix as a bioactive material to grow mature osteocytes. OB‐dECM was extracted from osteoblast cell sheets, decellularized, and finally reconstituted into a gel for embedding osteocytes (Figure S2). As a result, the viability of IDG‐SW3 osteocytes was enhanced when embedded in Col/OB‐dECM composite gel compared with those embedded in collagen gel (Figure 2a). However, gel shrinkage was observed at OB‐dECM concentrations of over 2 mg/ml, which limits long‐term cultivation in the bone‐on‐a‐chip (Figure 2b). Hence, the gel composite containing 2 mg/ml collagen and 1 mg/ml OB‐dECM was optimized for subsequent experiments. Furthermore, the viability of IDG‐SW3 cells also depended on the cell density (Figure S4). It was confirmed that the activity of IDG‐SW3 cells increased at a concentration of over 1 × 10^6^ cells/ml (Figure S4A), but gel shrinkage occurred at a concentration of 2 × 10^6^ cells/ml from Day 2 (Figure S4B,C). Therefore, the cell density in subsequent experiments was optimized to 1 × 10^6^ cells/ml. The Young's modulus and storage modulus were evaluated to determine whether OB‐dECM alters the mechanical strength of the gel composite. The Young's modulus (Figure 2c) and the storage modulus in the Col/OB‐dECM group were 7.2‐fold (*p* < 0.0001) and approximately threefold higher, respectively, than in the collagen group (Figure 2d), indicating that the Col/OB‐dECM group is stiffer than the collagen group. Generally, artificial bone models have used bone‐derived biomaterials to enhance the mechanical and functional characteristics of the matrix.^10^, ^31^ OB‐dECM has a relatively low mechanical strength compared with other artificial materials such as polymers, metals, and other composites. However, unlike the other materials, the OB‐dECM is a transparent matrix that allows for easy observation of cells and flexibility in biofabrication with spatial and temporal control of the gel, and it promotes cell functionality via bioactive components. These advantages of the material can offer a fast and effective way for high‐content screening (HCS) imaging and can be utilized as a useful alternative for building bone models.

### 3.3 Effect of OB‐dECM on osteogenesis in a bone‐on‐a‐chip

**FIGURE 3:**
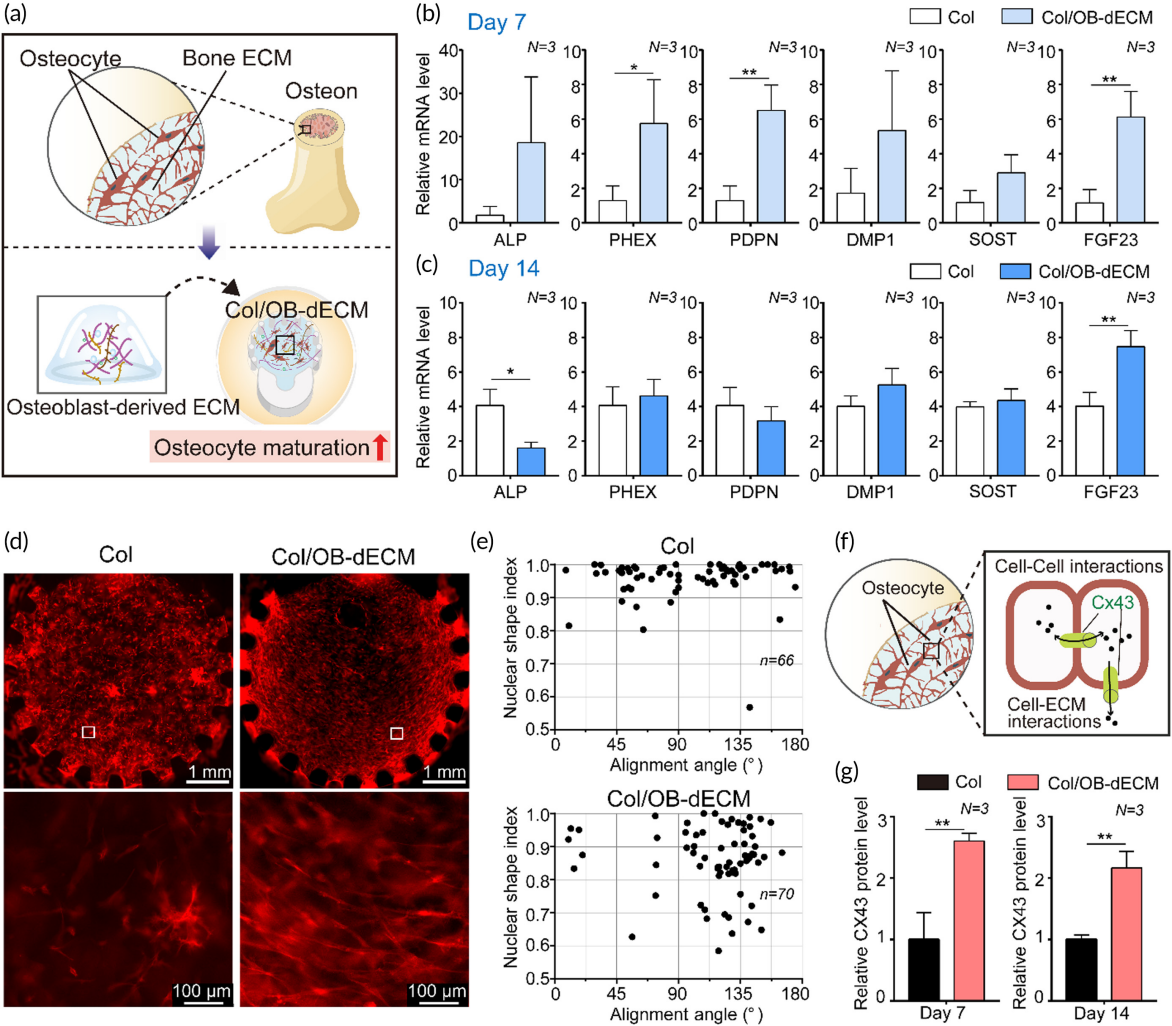
Effect of OB‐dECM on osteogenic differentiation and maturation of IDG‐SW3 cells in a bone‐on‐a‐chip. (a) A schematic showing the strategy for cell–ECM interaction using Col/OB‐dECM simulating an in vivo osteon. (b and c) Relative gene expression levels of early osteocyte differentiation markers (*ALP*, *PDPN*, and *PHEX*) and late osteocyte differentiation markers (*DMP1*, *SOST*, and *FGF23*) in IDG‐SW3 cells according to the gel type (collagen or Col/OB‐dECM). Assays were performed on Days 7 and 14 after differentiation of osteocytes within gel (*N* = 3). *GAPDH* was used as an internal control. (d) Morphological changes and alignment of IDG‐SW3 cells differentiated in collagen or Col/OB‐dECM hydrogels for 14 days (*N* = 3). The area enclosed in a white box in the images above was enlarged in the images below. The cells were immunostained against F‐actin (Alexa Fluor 594, red). (e) Nuclear shape index (NSI) and alignment angle of IDG‐SW3 cells analyzed using the representative confocal images (Figure S5) (Col, *n* = 66; Col/OB‐dECM, *n* = 70). (f) A schematic representing the implicit role of Cx43 on cellular function of osteocytes through cell–cell and cell–ECM interactions. (g) Cx43 protein level expressed in IDG‐SW3 cells according to the gel type. Assays were performed using ELISA on Days 7 and 14 after differentiation (*N* = 3). All values are expressed as mean ± SD (*p < 0.05, **p < 0.01). OB‐dECM, osteoblast‐derived decellularized extracellular matrix; ELISA, enzyme‐linked immunosorbent assay

Since the matrix surrounding the cells has an important role in maintaining bone strength and bone remodeling, the ECM for mimicking tissue niches is an attractive factor in the regulation of cell behaviors via cell–ECM interactions in tissue engineering. We hypothesized that OB‐dECM could promote the maturation of osteocytes (Figure 3a). When exposed to Col/OB‐dECM, early osteogenic markers, including a *ALP*, phosphate‐regulating endopeptidase homolog X‐linked (*PHEX*), and podoplanin (*PDPN*), and late osteogenic markers, including dentin matrix acidic phosphoprotein 1 (*DMP*1), sclerostin (*SOST*), and fibroblast growth factor 23 (*FGF23*) were upregulated in IDG‐SW3 cells on Day 7 (Figure 3b). *PHEX*, *PDPN*, and *FGF23* levels were significantly enhanced (*PHEX*, *p* < 0.05; *PDPN* and *FGF23*, *p* < 0.01). However, except for *ALP* and *FGF23*, the expression of other markers was not significantly different from those in the collagen group on Day 14 (Figure 3c). A decrease in the *ALP* level was observed (2.5‐fold, *p* < 0.05), which was consistent with previous reports stating that *ALP* level reduces as cell maturation proceeds.^24^ *FGF23* level was 1.9‐fold higher in the Col/OB‐dECM group (*p* < 0.01) than in the collagen group. This tendency indicated that the OB‐dECM can activate osteocyte differentiation in the early stages, although the sustained maturation was limited. One possible reason for this could be the limited functionality of the cell line.

Moreover, we observed a unique orientation of IDG‐SW3 cells when exposed to OB‐dECM, but this was hardly observed in the collagen group. Cells were concentrically arranged inside the Col/OB‐dECM group on Day 14, whereas they were randomly distributed in the collagen group (Figure 3d). This alignment was apparently stronger as IDG‐SW3 cells were more matured.

Based on previous studies regarding the cell alignment that affects the change in alignment and shape of the cell nucleus,^33^ we investigated changes in the NSI and alignment angle of the nucleus of IDG‐SW3 cells when exposed to OB‐dECM (Figures 3e and S5). The Col/OB‐dECM group had a lower NSI value than the collagen group, showing a stronger polarization of the nucleus. In addition, the nuclei with strong polarization were gathered around a similar alignment angle of 120° in the Col/OB‐dECM group. In contrast, the collagen group showed low polarization and no specific alignment angle of the nuclei. At this stage, the factor affecting this cell arrangement was not clearly identified, but with reference to the literature,^34^, ^35^ we focused on connexin 43 (Cx43), a well‐known gap junction protein and a key regulator of cell–cell and cell–ECM interactions via the gap junction channels and hemichannels (Figure 3f). Intracellular Cx43 levels in IDG‐SW3 cells of the OB‐dECM group were significantly increased by more than twofold (*p* < 0.01) compared with those in the collagen group (Figure 3g). It has been reported that osteocytes are major mechanosensory cells in bone tissue, and Cx43 at the cell surface plays a very important role in processing cell behavior between cells and extracellular environments.^36^

Based on these results, we confirmed that the use of a mixture of collagen and OB‐dECM could be more efficient in mimicking the in vivo osteon niche and in inducing osteocyte maturation compared with the use of collagen hydrogel only. At this stage, we did not identify the exact mechanisms of Cx43‐relevant cell orientation and the biochemical cues of OB‐dECM associated with cell–ECM interactions. Further investigation is necessary for our future studies.

### 3.4 Effect of coculture of osteocyte and osteoblast in a bone‐on‐a‐chip

During in vivo bone formation, osteocytes and osteoblasts communicate and precisely regulate each other for bone remodeling.^1^, ^3^, ^9^ The osteoblast layer on the surface of new bone plays a critical role in ECM production and its subsequent mineralization. Mature osteoblasts are known to be finally transformed into osteocytes.

**FIGURE 4:**
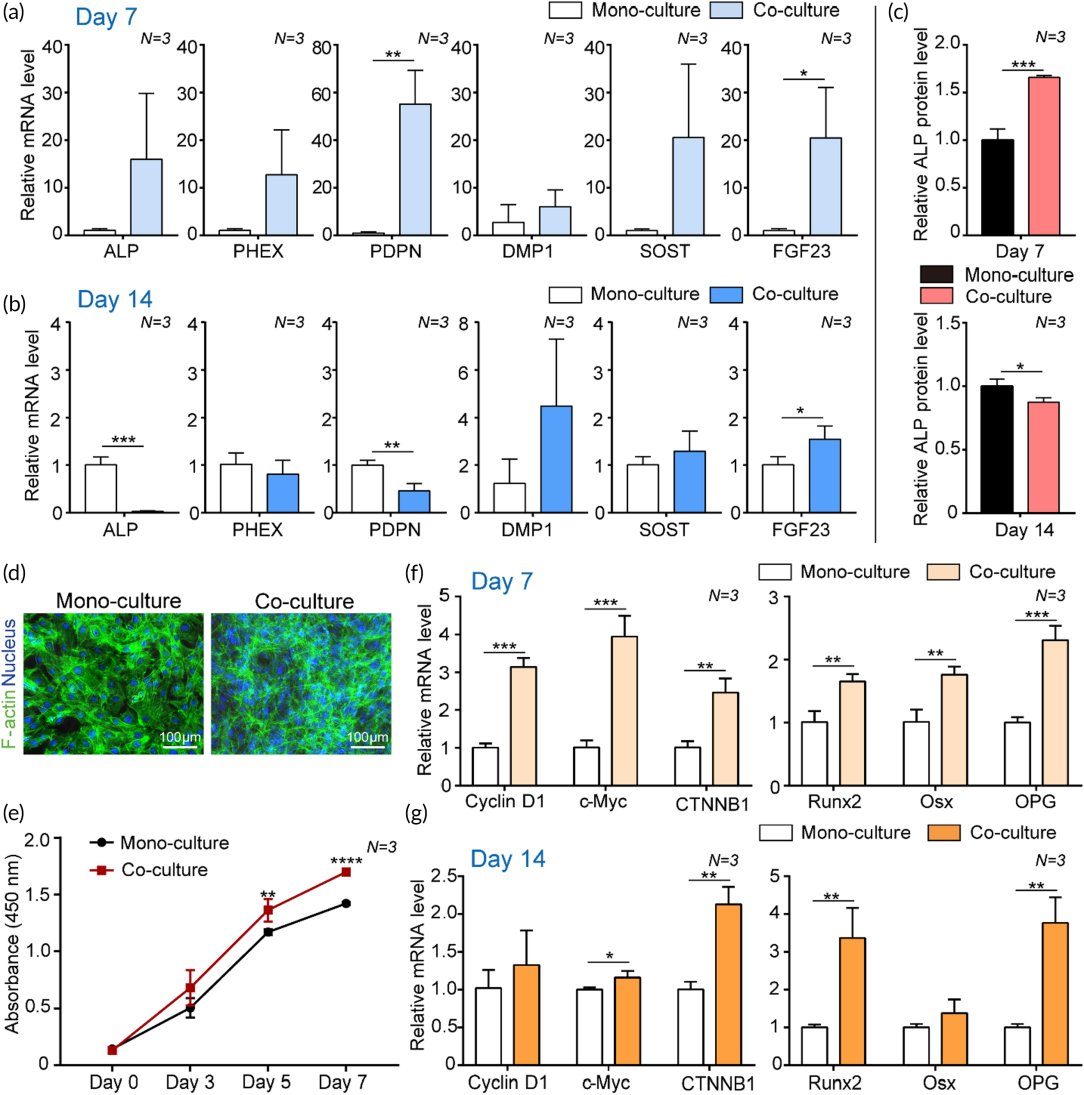
Effect of osteocyte and osteoblast coculture in a bone‐on‐a‐chip. (a–c) The osteocyte differentiation of IDG‐SW3 cells was affected upon coculture with MC3T3‐E1 cells. Relative gene expression levels of early differentiation markers (*ALP*, *PDPN*, and *PHEX*) and late differentiation markers (*DMP1*, *SOST*, and *FGF23*) in IDG‐SW3 cells monocultured or cocultured with MC3T3‐E1 cells on (a) Day 7 and (b) Day 14 after culture (*N* = 3). *GAPDH* was used as an internal control. (c) The ALP protein level of IDG‐SW3 cells in mono or cocultured groups measured using an ALP quantification assay on Day 7 and Day 14 after culture (*N* = 3). (d–g) The proliferation and osteoblastogenic differentiation of MC3T3‐E1 cells was affected upon coculture with IDG‐SW3 cells. (d) Representative immunofluorescence images showing staining against F‐actin (green) and nucleus (blue) of MC3T3‐E1 cells in monoculture or coculture groups on Day 7. (e) Proliferation rate of MC3T3‐E1 cells cocultured with IDG‐SW3 cells during 7 days measured using a CCK‐8 assay (*N* = 3). (f and g) Relative gene expression levels of proliferation markers (*Cyclin D1*, *c‐Myc*, and *CTNNB1*) and osteoblast differentiation markers (*Runx2*, *Osx*, and *OPG*) in MC3T3‐E1 cells monocultured or cocultured with IDG‐SW3 cells on (f) Day 7 and (g) Day 14 after culture (*N* = 3). *GAPDH* was used as an internal control. All values are expressed as mean ± SD (*p < 0.05, **p < 0.01, ***p < 0.001, ****p < 0.0001). CCK‐8, Cell Counting Kit‐8

Our bone‐on‐a‐chip platform has two different compartments inside and outside the gel chamber, allowing for cell–cell interactions through gaps between micropillars. In this study, we assumed that the coculture of cells could produce important key mediators of cell maturation and proliferation. We found that the levels of osteocyte markers in IDG‐SW3 cells were dramatically enhanced when cocultured on Day 7 (Figure 4a), especially *PDPN* (52.3‐fold, *p* < 0.01) and *FGF23* (20.0‐fold, *p* < 0.05). In mature osteocytes, the levels of early markers were significantly decreased (*ALP*: 34.3‐fold, *p* < 0.001; *PDPN*: 2.1‐fold, *p* < 0.01) on Day 14, whereas the late markers tended to increase in the coculture system (*FGF23*: 1.5‐fold, *p* < 0.05) (Figure 4b). These results implied that certain secretion factors of MC3T3‐E1 cells could promote the maturation of IDG‐SW3 cells. ALP protein level increased in the early stage (1.6‐fold, *p* < 0.001) and decreased in the late stage (1.1‐fold, *p* < 0.05) of differentiation of osteocytes (Figure 4c).

In the case of osteoblasts, osteocytes affected the proliferation and maturation of osteoblasts. First, the number of MC3T3‐E1 cells steadily increased in the coculture group for 7 days compared with that in the monoculture group (Figure 4d,e). On Days 5 and 7, a significant increase in cell number was observed in the coculture group. In addition, the expression levels of proliferation markers in MC3T3‐E1 cells were significantly upregulated in the coculture group (*cyclin D1*: 3.1‐fold, *p* < 0.001; *c‐Myc*: 3.9‐fold, *p* < 0.001; *CTNNB1*: 2.4‐fold, *p* < 0.01) compared with those in the monoculture group on Day 7 (Figure 4f). On Day 14, the expression levels of other proliferation markers were saturated, but the expression of *CTNNB1*, which encodes *β‐catenin*, significantly increased by 2.1‐fold (*p* < 0.01) (Figure 4g). In the coculture group, both proliferation and differentiation of MC3T3‐E1 cells were affected by IDG‐SW3 cells. On Day 7, the expression levels of differentiation markers of MC3T3‐E1 were upregulated (runt‐related transcription factor 2 [*RUNX2*]: 1.6‐fold, *p* < 0.01; osterix [*Osx*]: 1.7‐fold, *p* < 0.01; osteoprotegerin [*OPG*]: 2.3‐fold, *p* < 0.001) in the coculture group compared with those in the monoculture group (Figure 4f). On Day 14, the expression of Runx2 and OPG was still upregulated more than threefold, showing significant differences (*p* < 0.01) (Figure 4g). In the above results, the difference in the expression levels of proliferation markers between monoculture and coculture groups narrowed over time, but differences in differentiation levels widened over time. This appears to be a process in which MC3T3‐E1 cells, a preosteoblast cell type, become mature osteoblasts by enhancing osteoblastogenic differentiation following the initial rapid proliferation during coculture with IDG‐SW3 for 14 days. This indicates that the interaction between MC3T3‐E1 cells and IDG‐SW3 cells in bone‐on‐a‐chip can reflect the bone formation process occurring in bone in vivo.^6^

### 3.5 A comparison test with Transwell system

**FIGURE 5:**
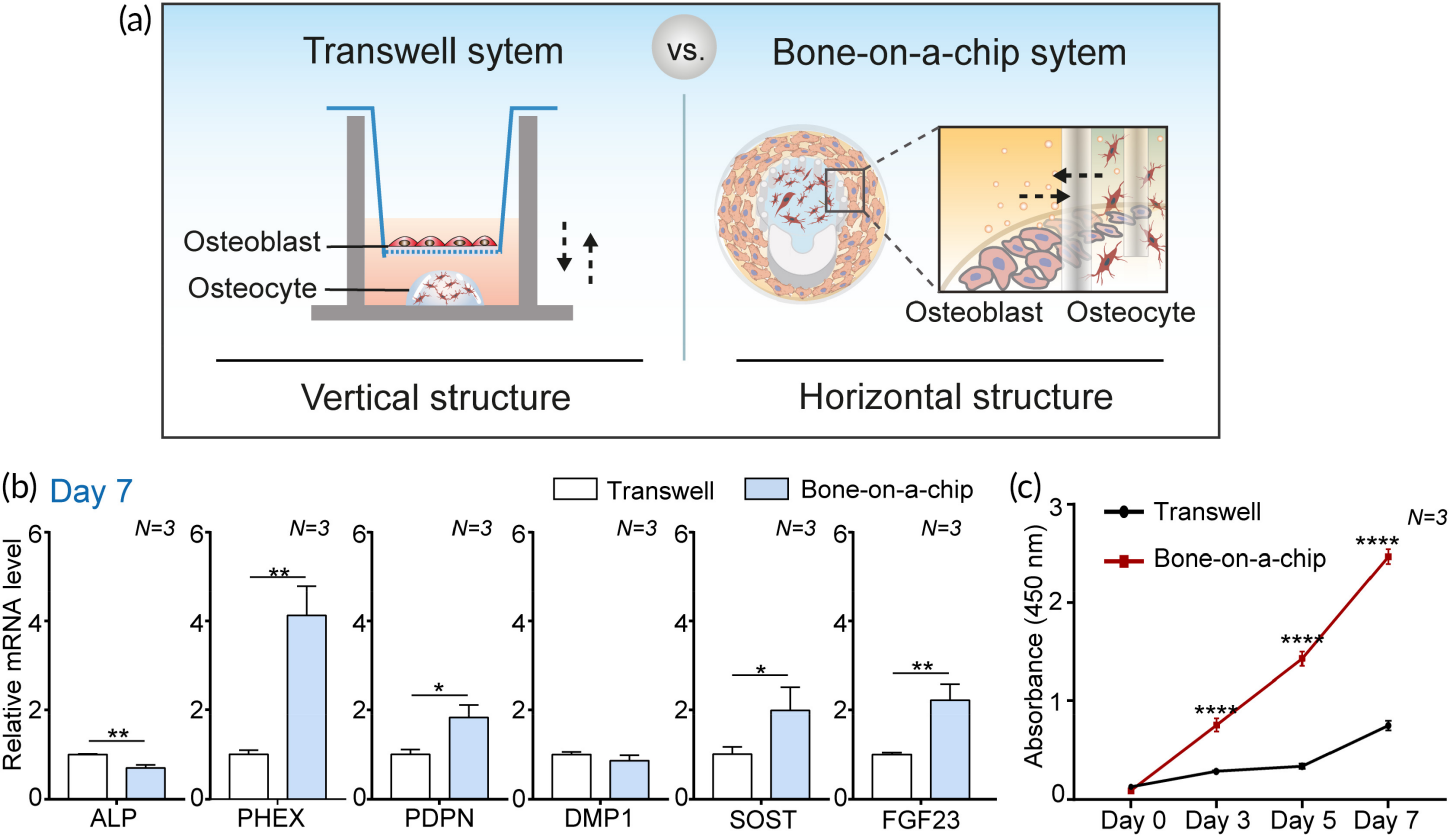
A comparison of the contribution of a bone‐on‐a‐chip and a Transwell system to bone maturation in osteocytes and osteoblasts. (a) Schematic illustration showing the difference in spatial structure between a bone‐on‐a‐chip and a Transwell system for coculturing osteocytes and osteoblasts. (b) Relative gene expression levels of early osteocyte markers (*ALP*, *PDPN*, and *PHEX*) and late osteocyte markers (*DMP1*, *SOST*, and *FGF23*) in IDG‐SW3 cells in each system on Day 7 (*N* = 3). IDG‐SW3 cells embedded in gel (1 × 10^4^ cells in 10 μl gel) were cocultured with osteoblasts (500 cells/well). All conditions were the same in both systems. *GAPDH* was used as an internal control. (c) Proliferation rate of MC3T3‐E1 cells cocultured with IDG‐SW3 cells during 7 days measured using a CCK‐8 assay (*N* = 3). All values are expressed as mean ± SD (*p < 0.05, **p < 0.01, ****p < 0.0001). CCK‐8, Cell Counting Kit‐8

As a high‐throughput system for drug testing, our platform has many advantages over traditional platforms, such as a Transwell system, in which cell culture compartments are vertically arranged. Our bone‐on‐a‐chip system has two horizontally arranged compartments for culturing two different types of cells, which greatly contributes to the ease of cell analysis (Figure 5a). As the focal planes for each cell are similar along the *z*‐axis, it is easy to observe both cells simultaneously and to observe cell–cell interactions at the border of the region where the two types of cells meet.

In addition to these advantages, we confirmed that our bone‐on‐a‐chip system is superior to the Transwell system with respect to the enhancement of cell functionality (Figure 5a–c). To achieve this goal, two types of cells were cocultured in each system, as shown in Figures 5a and S2B. IDG‐SW3 cells were placed in the gel, and MC3T3‐E1 cells were placed in the region surrounding the IDG‐SW3 cells. This cell placement simulated the osteon configuration. All culture conditions, such as cell number, gel volume, and medium volume, were the same in both systems. The expression levels of osteocyte differentiation markers in the bone‐on‐a‐chip were significantly upregulated (*PHEX*: 4.1‐fold, *p* < 0.01; *PDPN*: 1.8‐fold, *p* < 0.05; *SOST*: 2.0‐fold, *p* < 0.05; *FGF23*: 2.2‐fold, *p* < 0.01) compared with those in the Transwell group on Day 7 (Figure 5b). In addition, considerable enhancement of MC3T3‐E1 cell proliferation was observed for 7 days in the bone‐on‐a‐chip compared with that in the Transwell system (Figure 5c). Accumulating evidence indicates that physical contact between osteocytes and osteoblasts is essential in the dynamic control of cell–cell interaction and terminally regulates bone differentiation and bone homeostasis in vivo.^9^, ^37^, ^38^ Moreover, the direct cell–cell communication via gap junctions has been investigated as one of the strong mechanisms.^9^, ^37^ The availability of a direct interaction between cells at the border area of the gel chamber and the relatively shorter distance between each cell region in our platform might efficiently promote cell–cell interactions. These results indicate that the configurational mimicking of in vivo bone in the bone‐on‐a‐chip led to biologically positive and synergistic effects.

### 3.6 Osteoporosis drug testing in a bone‐on‐a‐chip

For practical use, a standardized and reliable system for obtaining reproducible data from hundreds of samples is equally important as recreating a physiologically relevant model. In addition to biomimetic functionality, this bone‐on‐a‐chip platform is easy to handle and can be applied to various well‐established well plate‐based instruments, enabling high‐throughput analysis.

**FIGURE 6:**
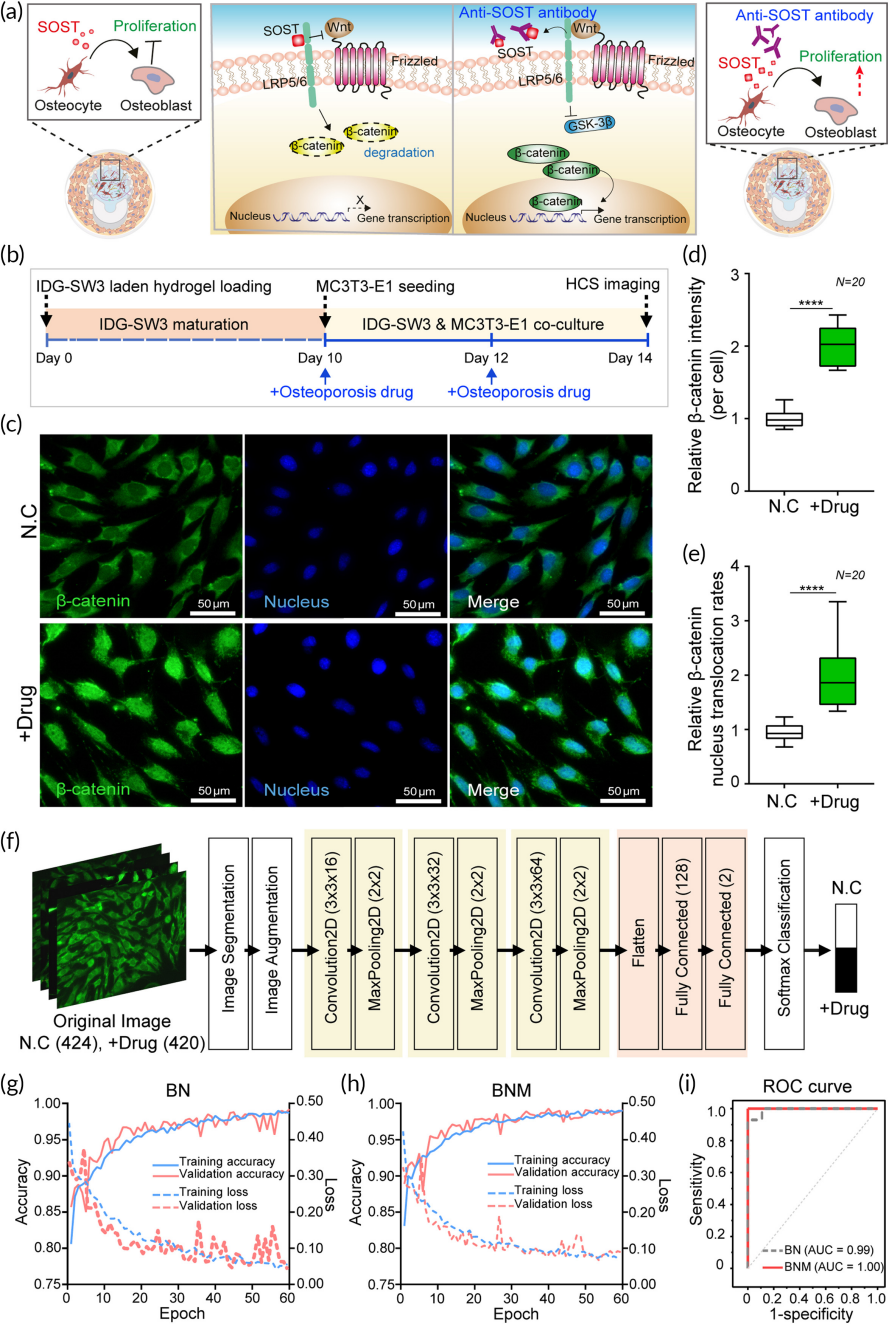
Osteoporosis drug testing using bone‐on‐a‐chip. (a) A schematic showing the Wnt pathway related to bone formation, one of the target mechanisms for osteoporosis treatment. SOST, secreted by osteocytes, downregulates osteoblast proliferation (left image) whereas an anti‐SOST antibody (used as an osteoporosis drug) upregulates osteoblast proliferation through nuclear translocation of β‐catenin via the Wnt pathway (right image). (b) The overall sequential process for osteoporosis drug treatment in the bone‐on‐a‐chip. After 10 days of IDG‐SW3 maturation for SOST secretion, MC3T3‐E1 cells were cocultured for additional 4 days. Cells were treated twice with an osteoporosis drug on Days 10 and 12. (c) Representative images of MC3T3‐E1 cells in osteoporosis drug‐treated and untreated groups that were immunostained against β‐catenin (green) and nucleus (blue). (d) Relative average fluorescence intensity of β‐catenin in cells in each drug‐treated group compared to that in the control (*N* = 20). (e) Relative β‐catenin nuclear translocation rate in drug‐treated group compared to that in the control (*N* = 20). (f) The proposed CNN‐based deep learning architecture to perform osteoporosis drug testing. (g and h) Accuracy (left *x*‐axis) and loss curves (right *x*‐axis) of CNN training and validation for the (g) BN and (h) BNM datasets with successive epochs. (i) ROC curve analysis comparing classification results in BN and BNM datasets. AUC, area under the ROC curve; BN, β‐catenin and nucleus fluorescence image data; BNM, β‐catenin, nucleus, and their merged image data; CNN, convolutional neural network; ROC, receiver operating characteristic

In the present study, we verified whether our bone‐on‐a‐chip system could be utilized for drug testing for osteoporosis. Osteoporosis is one of the most common diseases in postmenopausal women, and various treatments and drugs have been extensively studied with several related mechanisms.^1^, ^4^ Among them, osteoblast formation‐associated drugs have attracted attention for osteoporosis treatment in recent years.^4^, ^39^ These drugs target sclerostin (SOST) protein, a negative bone mass regulator which is secreted by osteocytes as a soluble signaling molecule, and works mainly by increasing new bone formation.^39^ As depicted in Figure 6a, SOST secreted by osteocytes is transferred to osteoblasts, which subsequently inhibits the Wnt signaling pathway through β‐catenin degradation.^5^, ^39^, ^40^ Using this principle, osteoporosis drugs have been developed as monoclonal antibodies that effectively bind to a specific region of SOST, which consequently blocks the binding between SOST and LRP5/6. These drugs ultimately promote β‐catenin nuclear translocation and activation of the transcriptional pathway into bone formation through osteoblasts.^39^, ^40^ Considering the remarkable effects of this drug, called romosozumab (trade name Evenity), on bone formation and inhibition of osteoporosis, it was recently approved by FDA in 2019.

In this respect, the bone‐on‐a‐chip system, where osteoblasts and osteocytes interact and regulate each other in the osteon‐like microenvironment, can be a suitable model for evaluating osteoporosis drugs. To demonstrate this conceptually, we verified whether the monoclonal SOST antibodies affect the bone formation similar to an osteoporosis drug in our bone‐on‐a‐chip system. To determine the concentration of the antibody (hereafter, osteoporosis drug) for treatment, the amount of SOST released from IDG‐SW3 cells was quantified using ELISA and finally fixed to 20 ng/ml in cell medium (Figure S6). This result showed that the SOST level in IDG‐SW3 cells increased as the cells matured, and the level rapidly increased on Day 10. Accordingly, we seeded the MC3T3‐E1 cells and treated them with the osteoporosis drug on Day 10 after the prematuration of IDG‐SW3 cells. Next, we assayed the response of the MC3T3‐E1 cells to SOST on Day 14 (Figure 6b). The osteoporosis drug did not affect the proliferation of MC3T3‐E1 cells monocultured in our platform (Figure S7) because there were no osteocytes that secrete SOST. This indicates that the microenvironment in the bone‐on‐a‐chip system, where cells regulate each other, is valid for testing the osteoporosis drug.

With the ease of observing the cell morphological phenotypes in a high‐throughput system, we evaluated the efficacy of this drug in the bone‐on‐a‐chip. β‐Catenin, whose degradation is blocked by osteoporosis drugs and nuclear translocation via the Wnt/β‐catenin pathway, can be used as a useful indicator to confirm drug efficacy. In immunofluorescence images, β‐catenin localization in MC3T3‐E1 cells cocultured with IDG‐SW cells was analyzed after treatment with the osteoporosis drugs (Figure 6c). In the nontreated group, β‐catenin was mainly distributed in the cytoplasm of cells, whereas it was mainly found in the nucleus in the drug‐treated group. These results correspond to the quantitative results in terms of the intensity and nuclear translocation rates of β‐catenin based on images obtained from multiple wells (20 wells) of the bone‐on‐a‐chip platform. As a result, the drug‐treated group had 2.0‐fold higher (*p* < 0.0001) intensity of β‐catenin compared with that in control group (Figure 6d). In addition, the drug‐treated groups had a 2.1‐fold higher (*p* < 0.0001) β‐catenin nuclear translocation rate compared with that in the control group (Figure 6e). The detailed methods for quantifying β‐catenin nuclear translocation are shown in Figure S3. These results show that osteoporosis drug evaluation based on image analysis could work well with the bone‐on‐a‐chip platform and also indicate that this platform could be useful when combined with an automatic HCS imaging system.

### 3.7 AI‐powered image analysis for osteoporosis drug testing

The assessment of drug efficacy through image analysis is sometimes difficult because the criteria for evaluation vary depending on the experimental setting selected and the experimental conditions, leading to nonreproducible and incorrect results. To overcome this difficulty, we introduced a deep learning algorithm based on CNNs into the imaging analysis with several hundreds of images obtained from our high‐throughput bone chips.^41^ We extracted green (β‐catenin), blue (nucleus), and merged channels from the original RGB images in the drug‐treated group and in the nondrug group, after treatment of cells with the osteoporosis drug. To develop a highly efficient deep learning algorithm, image segmentation and augmentation were performed to increase the input image data.^42^ Based on CNNs involving convolution layers, pooling layers, and fully connected layers (Table S2), an algorithm was developed and the overall process of the algorithm to classify drug‐treated and nontreated groups is shown in Figure 6f.

The proposed deep learning modality was trained using two different datasets: a dataset of β‐catenin and nuclear images (BN), and a dataset of β‐catenin and nuclear and merged images (BNM). The neural network was trained for 60 epochs. A 10‐fold cross‐validation technique was used to measure the performance of the model. This procedure was repeated for each classification problem. The results showed the loss and accuracy of the classification algorithm in training and validation using the BN dataset (Figure 6g) and BNM dataset (Figure 6h). The final accuracy of the algorithm in the test set was 97.2% and 99.5% in the BN and BNM datasets, respectively (Table S3). This drug evaluation algorithm had high accuracy in both datasets and was not significantly but slightly higher in accuracy when using the BNM datasets. This difference might be attributed to the increased information obtained from the images used for training the network. Figure 6i shows the ROC curves to show the diagnostic ability of the drug evaluation algorithm. The high accuracy and AUC of the classification algorithm using BN (accuracy = 97.2%, AUC = 0.99) and BNM (accuracy = 99.5%, AUC = 1.00) were obtained. This indicates that the model is a stable and efficient with high accuracy for osteoporosis drug testing.

### 3.8 Current prospects and challenges of the developed bone‐on‐a‐chip

The developed bone‐on‐a‐chip integrated with a high‐throughput well plate can simulate physiological phenomena in bones. A vast amount of image data obtained from this platform contributes to the development of a deep learning algorithm that can be used to evaluate osteoporosis drugs. Adopting the same concept, other potential target markers or target diseases could be suggested for evaluation. The algorithms could also be greatly improved by increasing the number of images or by varying the additional information when evaluating bone disease‐related drugs.^43^

Although this study focused on the relationship between osteoblasts and osteocytes, osteoclasts are also important factors in bone remodeling based on the bone resorption‐related osteoporosis mechanism. Therefore, we need to further investigate the osteocyte–osteoblast–osteoclast interactions in our platform by simultaneously culturing all three of them, and this will provide us with much more insights into the regulation of bone formation and bone resorption process of bone metabolism for the treatment of osteoporosis. Romosozumab, one of the most prominent osteoporosis drugs, has been reported to have dual effects of causing not only an increase in bone formation but also a decrease in bone resorption.^44^ Although this drug associated with SOST binding has powerful effects on bone mass regulation,^31^, ^39^, ^45^, ^46^ it still has a cardiovascular risk.^45^, ^46^ Therefore, the demand for the development of new drugs and the need for continuous efforts still exist. Our bone‐on‐a‐chip platforms will be useful in evaluating the performance and side effects of potential drugs in preclinical studies.

## 4 CONCLUSION

In this study, we developed a biomimetic bone model with physically and biologically relevant characteristics using a combination of top‐down and bottom‐up approaches. OB‐dECM contains bioactive components and induces cell–ECM interactions, and consequently enhances osteogenic functionality in osteoblasts and osteocytes. In addition, coculture synergistically promoted osteogenic and osteoblastogenic differentiation and maturation in a microstructured 3D cell unit. More importantly, the standardized configuration of bone units built in a well plate can provide uniform and multiple units of in vitro bone models, enabling simultaneous and high‐throughput drug testing and analysis. We analyzed the intelligent deep learning algorithm using a vast amount of image data obtained from this platform, and validated its feasibility for osteoporosis drug testing. We can conclusively state that this platform can open up new opportunities for precise, cost‐ and time‐efficient methodologies in preclinical studies concerning the pharmaceutical field.

## AUTHOR CONTRIBUTIONS

**Kyurim Paek:** Conceptualization (supporting); data curation (equal); formal analysis (equal); methodology (equal); writing – original draft (equal). **Seulha Kim:** Data curation (equal); formal analysis (supporting); methodology (equal); resources (equal). **Sungho Tak:** Data curation (equal); formal analysis (supporting); methodology (equal); software (equal). **Min Kyeong Kim:** Data curation (supporting); visualization (lead); writing – review and editing (supporting). **Jubin Park:** Formal analysis (supporting); visualization (supporting). **Seok Chung:** Investigation (supporting); writing – review and editing (supporting). **Tai Hyun Park:** Investigation (supporting); resources (equal). **Jeong Ah Kim:** Conceptualization (lead); funding acquisition (lead); investigation (lead); project administration (lead); resources (equal); supervision (lead); writing – original draft (equal); writing – review and editing (equal).

## CONFLICTS OF INTERESTS

The authors declare no conflicts of interest.

### PEER REVIEW

The peer review history for this article is available at https://publons.com/publon/10.1002/btm2.10313.

## Supporting information


**Appendix S1** Supporting Information

## Data Availability

The data that supports the findings of this study are available in the supplementary material of this article.
