# Supplementary material for: A high‐throughput biomimetic bone‐on‐a‐chip platform with artificial intelligence‐assisted image analysis for osteoporosis drug testing
Source: Bioeng Transl Med. 2022 Apr 5;8(1):e10313. doi: 10.1002/btm2.10313 (PMC9842054; doi:10.1002/btm2.10313)
Supplement: Supplementary file 1 — Appendix S1 Supporting Information [file BTM2-8-e10313-s001.docx]

Supporting Information

**A high-throughput biomimetic bone-on-a-chip platform with artificial intelligence-assisted image analysis for osteoporosis drug testing**

Kyurim Paek^1,2^, Seulha Kim^3^, Sungho Tak^4^, Min Kyeong Kim^1^, Jubin Park^1,2^, Seok Chung^2,5^, Tai Hyun Park^3^, and Jeong Ah Kim^1,6*^

*^1^Center for Scientific Instrumentation, Korea Basic Science Institute, Daejeon 34133, Republic of Korea*

*^2^Program in Micro/Nano System, Korea University, Seoul 02841, Republic of Korea*

*^3^School of Chemical and Biological Engineering, Institute of Chemical Processes, Seoul National University, Seoul 08826, Republic of Korea*

*^4^Research Center for Bioconvergence Analysis, Korea Basic Science Institute, Cheongju, Chungbuk 28119, Republic of Korea*

*^5^School of Mechanical Engineering, Korea University, Seoul 02841, Republic of Korea*

*^6^Department of Bio-Analytical Science, University of Science and Technology, Daejeon 34113, Republic of Korea*

***Corresponding author:**

J. A. Kim, Tel: +82 43 240 5068; Fax: +82 43 240 5158; Email: jakim98@kbsi.re.kr.

**Supplementary tables:**

**Table S1. List of primer sequence for qRT-PCR.**

| Gene name | Forward primer sequences (5’-3’) | Reverse primer sequences  (5’-3’) | Accession number |
| --- | --- | --- | --- |
| *GAPDH* | GCATCCTGCACCACCAACTGCTT | TCTTCTGGGTGGCAGTGATGGCA | NM_001289726.1 |
| *ALP* | GCACTGCCACTGCCTACTTGTGT | AGCGCAGGATGGATGTGACCTCA | NM_007431.3 |
| *PHEX* | TGAGACCAGCCACCAAACCACGA | ACAGGGCCAGTGCAATCCGAATG | NM_011077.2 |
| *PDPN* | ACCGTGCCAGTGTTGTTCTG | AGCACCTGTGGTTGTTATTTGT | NM_010329.3 |
| *DMP1* | AGCCCACGAGCACTCAGGATTCA | TCGCTCTGGGTTTCCCTGCTGTT | NM_001359013.1 |
| *SOST* | AGCCTCCTCCTGAGAACAACCAG | CTCGGACACATCTTTGGCGTCAT | NM_024449.6 |
| *FGF23* | AGAGGACGCCGGCTCTGTGG | CGGCCCAGGCTCACCAGGTA | NM_022657.4 |
| *Cyclin D1* | CCTTGACTGCCGAGAAGTTG | AGTTCCATTTGCAGCAGCTC | NM_001379248.1 |
| *c-Myc*^1^ | TCCTGTACCTCGTCCGATTC | GGTTTGCCTCTTCTCCACAG | NM_010849.4 |
| *CTNNB1*^1^ | ATGGCTTGGAATGAGACTGC | ATGCTCCATCATAGGGTCCA | NM_007614.3 |
| *Runx2*^2^ | AAGTGCGGTGCAAACTTTCT | ACGCCATAGTCCCTCCTTTT | NM_001146038.2 |
| *Osterix* | AGGAAGAAGCTCACTATGGC | AGTCCATTGGTGCTTGAGAA | NM_130458.4 |
| *OPG*^3^ | AAAGCACCCTGTAGAAAACA | CCGTTTTATCCTCTCTACACTC | NM_008764.3 |

**Table S2. The output of the proposed network architecture.**

| Layer (type) | Output shape | Parameter # |
| --- | --- | --- |
| Conv2d_1 (Conv2D) | (None, 180, 180, 16) | 448 |
| Max_pooling2d_1 (MaxPooling2D) | (None, 90, 90, 16) | 0 |
| Conv2d_2 (Conv2D) | (None, 90, 90, 32) | 4640 |
| Max_pooling2d_2 (MaxPooling2D) | (None, 45, 45, 32) | 0 |
| Conv2d_3 (Conv2D) | (None, 45, 45, 64) | 18496 |
| Max_pooling2d_3 (MaxPooling2D) | (None, 22, 22, 64) | 0 |
| Dropout (Dropout) | (None, 22, 22, 64) | 0 |
| Flatten_1 (Flatten) | (None, 30976) | 0 |
| Dense_1 (Dense) | (None, 128) | 3965056 |
| Dense_2 (Dense) | (None, 2) | 258 |
| Total parameters: 3,988,898  Trainable parameters: 3,988,898  Non-trainable parameters: 0 |  |  |

**Table S3. Performance of the classification algorithm using different types of image data sets.**

| Image type | Test | |
| --- | --- | --- |
|  | Loss | Accuracy |
| BN | 0.094 | 0.972 |
| BNM | 0.019 | 0.995 |

**BN:** Dataset of β-catenin and nuclear images; **BNM:** Dataset of β-catenin and nuclear and merged images

**Supplementary figures**

**
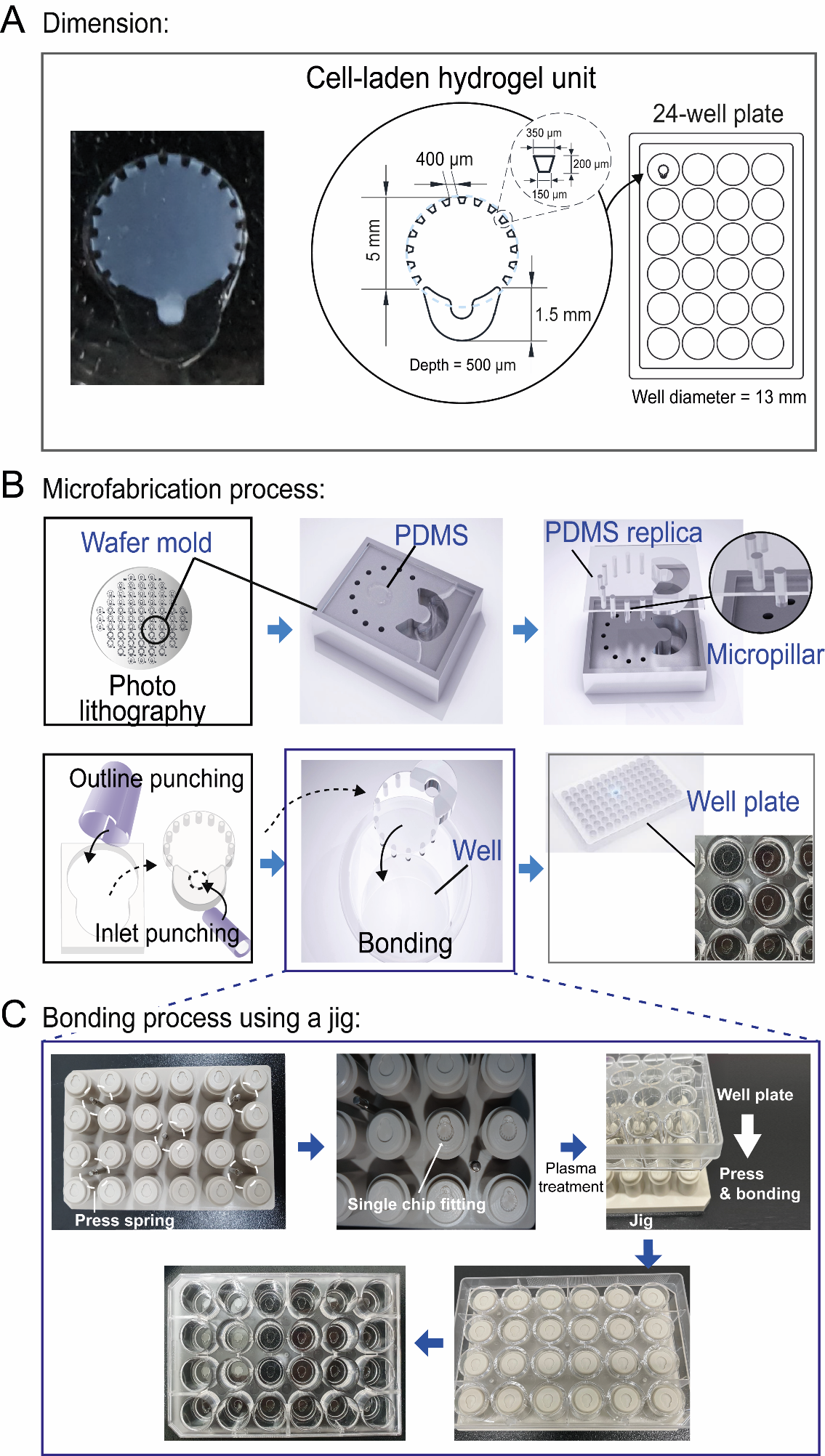
**

**Figure S1. Fabrication process of bone-on-a-chip platform.** (A) Design of a bone-chip unit for incorporating cell-laden hydrogel, which is integrated in a single well within a 24-well plate. (B) Schematic showing the overall fabrication process of the bone-on-a-chip platform. This process involves conventional photolithography and soft lithography using PDMS.^4^ (C) A detailed depiction of the advanced bonding process. The jig was applied to ensure accurate centering of the chips within each well for high-throughput analysis.

**
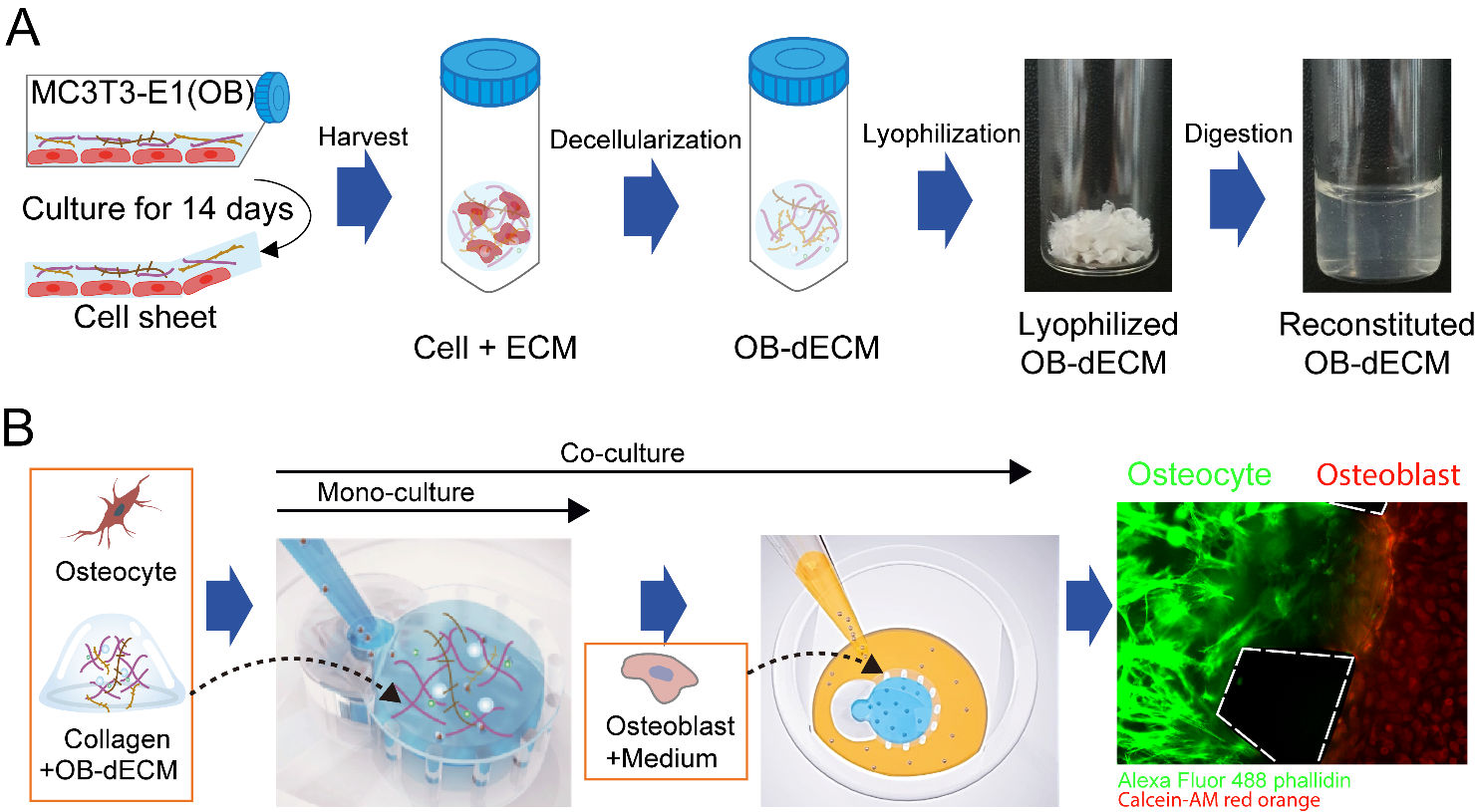
**

**Figure S2. Col/OB-dECM hydrogel for culturing bone cells.** (A) Overall preparation process of OB-dECM derived from MC3T3-E1 cells.^5^ (B) Co-culture process of IDG-SW3 and MC3T3-E1 cells in a bone-on-a-chip. IDG-SW3-laden Col/OB-dECM hydrogels were loaded in a chip chamber. MC3T3-E1 cells maintained in osteogenic medium were simultaneously added around the chip within a well for co-culture. In mono-culture, the osteogenic medium was added without cells.

**
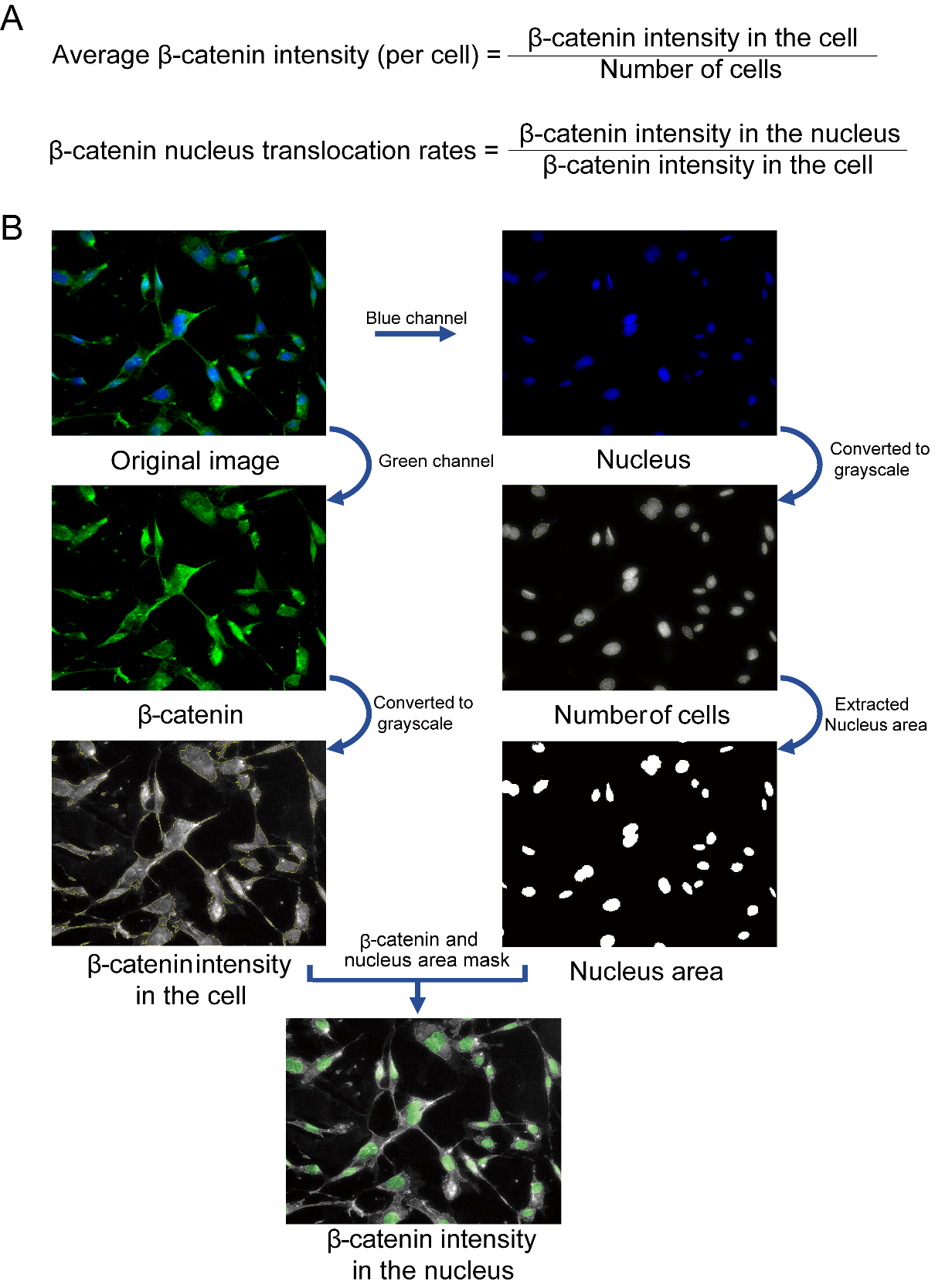
**

**Figure S3.** **Image analysis procedure for the measurement of β-catenin intensity and translocation rates in MC3T3-E1 cells after osteoporosis drug testing.** (A) Equations for calculating average β-catenin intensity (per cell) and β-catenin nuclear translocation rates. (B) The stepwise process of image analysis method for calculating average β-catenin intensity (per cell) and β-catenin nuclear translocation rate.


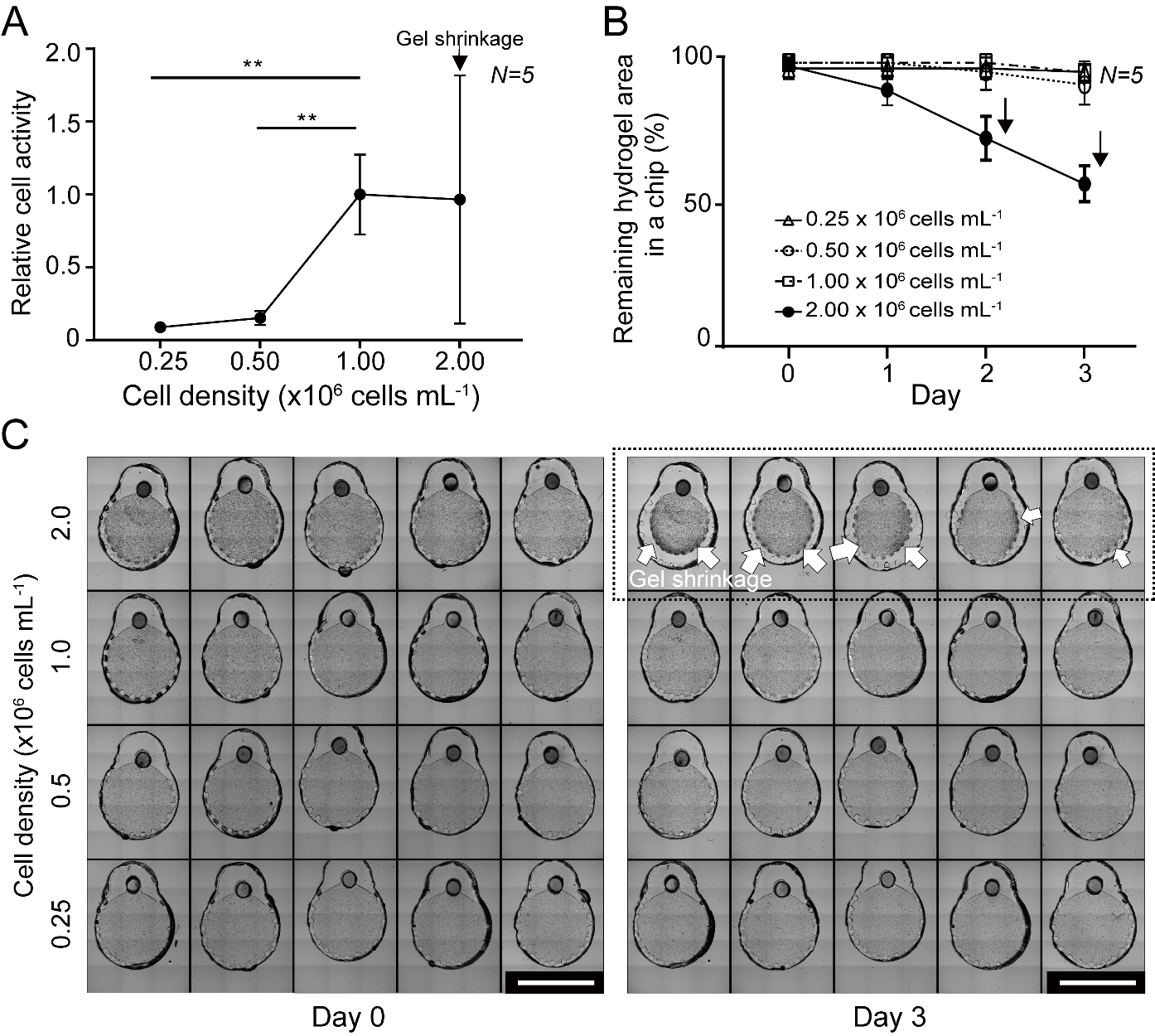


**Figure S4. Optimization of IDG-SW3 cell density.** Optimization of cell seeding density in a bone-on-a-chip unit. (A) Relative cell activity at different cell densities in hydrogels performed on day 3 after cell seeding was analyzed using 3D cell viability assay kit (*N=5*). (B) Remaining hydrogel area (%) in a chip relative to original gel area after gel shrinkage according to different cell densities during 3 days (*N=5*). (C) Images of hydrogel shrinkage according to different cell densities on days 0 and 3. Scale bar represents 5 mm. All values are expressed as mean ± S.D. (** *p* < 0.01).


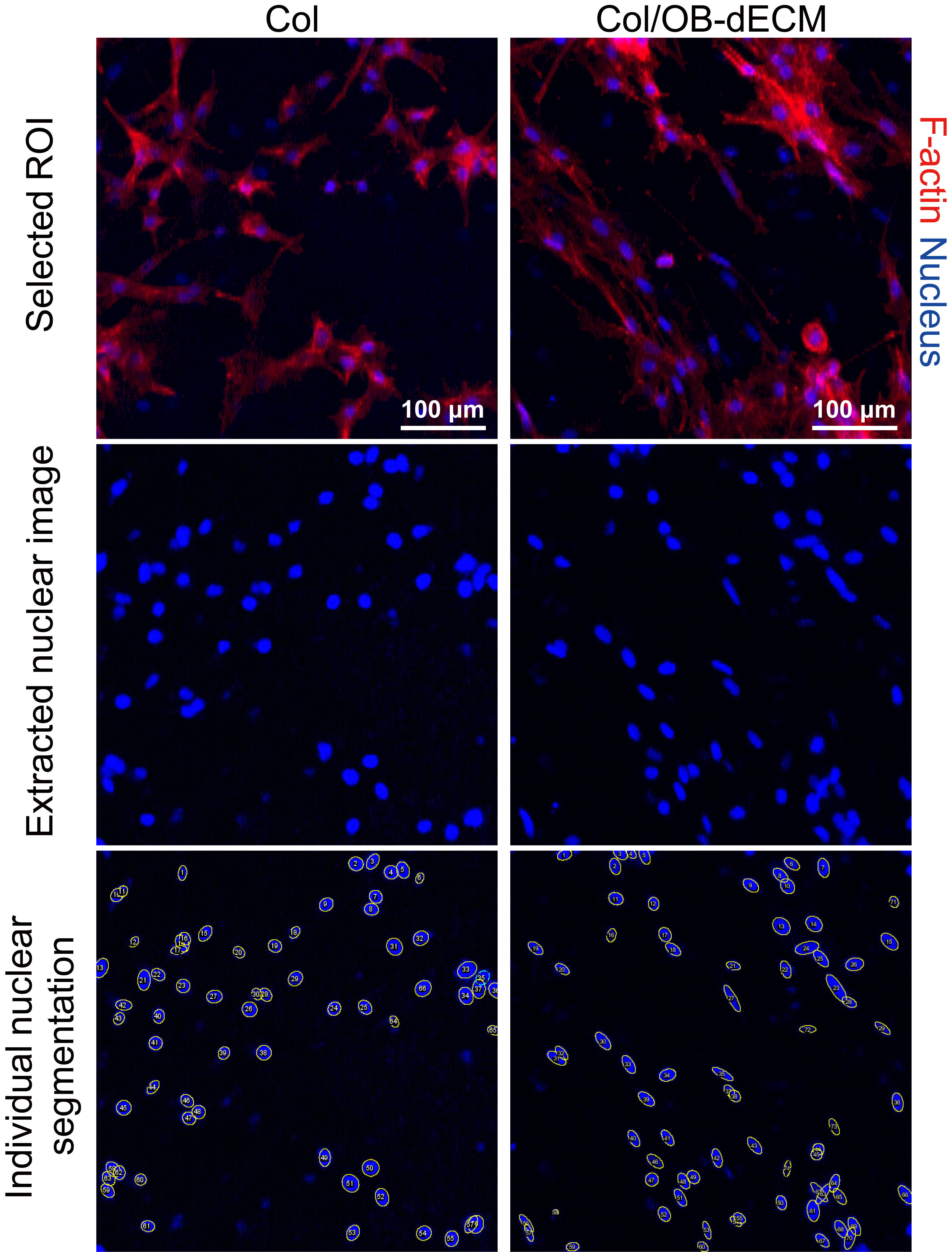


**Figure S5.** **Image analysis procedure for the nuclear shape index (NSI) and nuclear alignment angle of IDG-SW3 cells in hydrogels.** Region of interest (ROI) was selected from fluorescence images of IDG-SW3 cells in the hydrogels. Nuclear images were subsequently extracted from the ROI. After separating each nucleus from the extracted nuclear image, the perimeter, area, and angle of the nucleus were calculated, and nuclear shape analysis was performed.


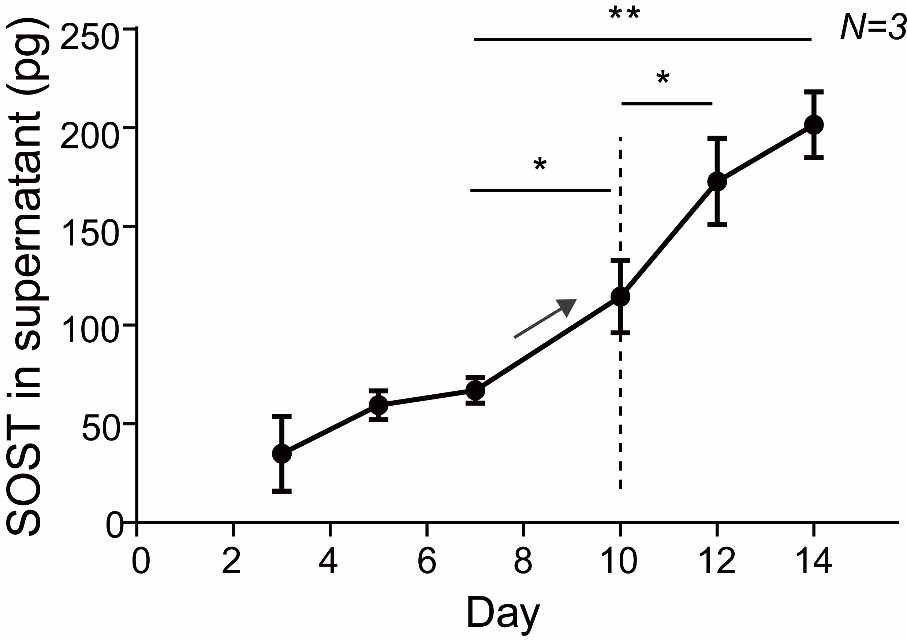


**Figure S6.** **Measurement of SOST secreted from IDG-SW3 cells in a bone-on-a-chip.** The amount of SOST secreted from IDG-SW3 cells cultured in a bone-on-a-chip was determined using ELISA at each time point during 14 days (*N=3*). Total SOST per well was quantified during the culture period. All values are expressed as mean ± S.D. (* *p* < 0.05, ** *p* < 0.01).


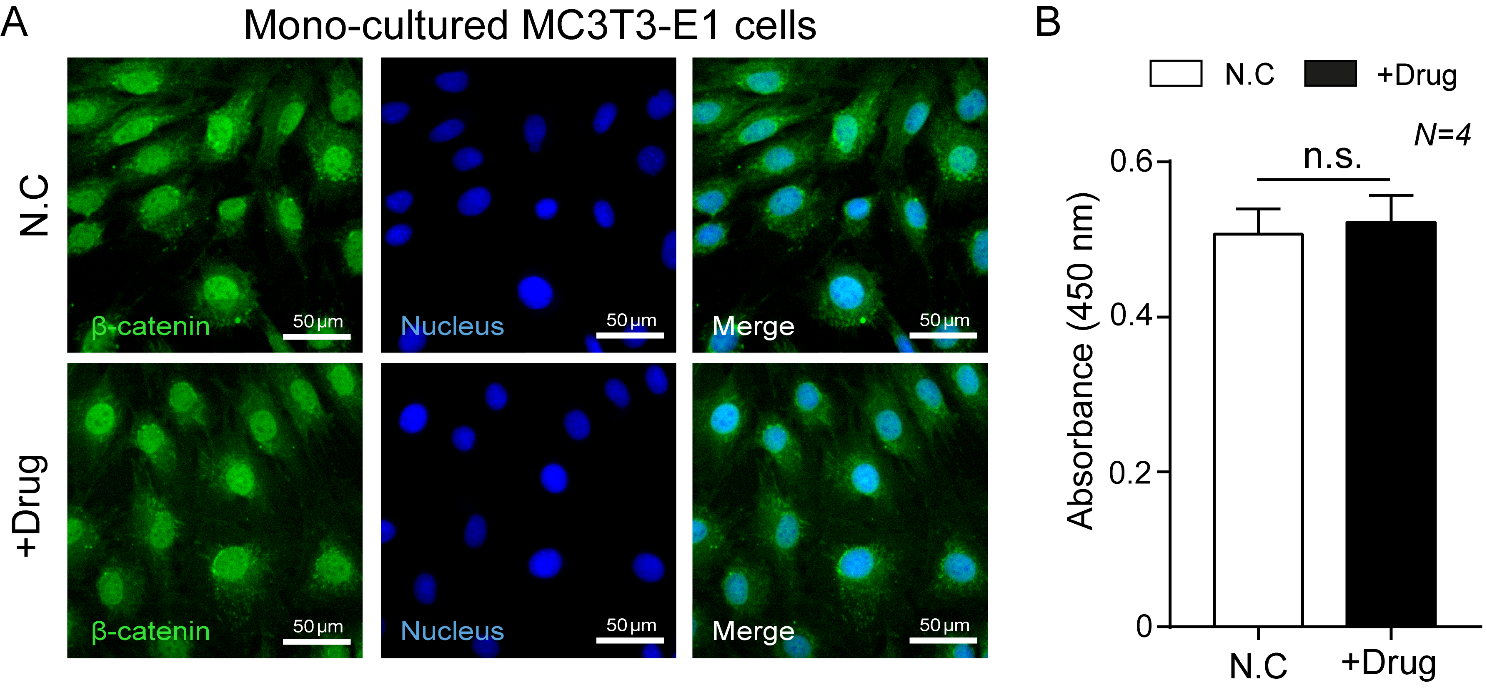


**Figure S7.** **Osteoporosis drug testing on mono-cultured osteoblasts.** MC3T3-E1 cells were seeded outside the gel region (5×10^2^ cells/ well) and mono-cultured without IDG-SW3 cells (no cells in the central gel). Cells were left untreated or were treated with osteoporosis drug (20 ng mL^-1^) and cultured for 4 days. (A) Representative immunostained images (β-catenin, green) of drug-treated and untreated groups on day 4. (B) Change in the proliferation of MC3TE-E1 cells upon treatment of the mono-culture with the drug. CCK-8 assay was performed on day 4. All values are expressed as mean ± S.D. (n.s., not significant).

**References**

1. Mei G, Zou Z, Fu S, et al. Substance P activates the Wnt signal transduction pathway and enhances the differentiation of mouse preosteoblastic MC3T3-E1 cells. *Int J Mol Sci*. 2014;**15**(4):6224-6240.

2. Hayashi K, Yamaguchi T, Yano S, et al. BMP/Wnt antagonists are upregulated by dexamethasone in osteoblasts and reversed by alendronate and PTH: potential therapeutic targets for glucocorticoid-induced osteoporosis. *Biochem Biophys Res Commun*. 2009;**379**(2):261-266.

3. Bendre MS, Montague DC, Peery T, Akel NS, Gaddy D, Suva LJ. Interleukin-8 stimulation of osteoclastogenesis and bone resorption is a mechanism for the increased osteolysis of metastatic bone disease. *Bone*. 2003;**33**(1):28-37.

4. Yu YJ, Kim YH, Na K, et al. Hydrogel-incorporating unit in a well: 3D cell culture for high-throughput analysis. *Lab Chip*. 2018;**18**(17):2604-2613.

5. Kim S, Lee SS, Son B, Kim JA, Hwang NS, Park TH. Partially Digested Osteoblast Cell Line-Derived Extracellular Matrix Induces Rapid Mineralization and Osteogenesis. *ACS Biomater Sci Eng*. 2021;**7**(3):1134-1146.
